# Hydrogenation of crude and purified d-glucosone generated by enzymatic oxidation of d-glucose

**DOI:** 10.1039/d0ra05512c

**Published:** 2020-08-18

**Authors:** Robert Lassfolk, Atte Aho, Dmitry Yu. Murzin, Reko Leino

**Affiliations:** Laboratory of Molecular Science and Technology, Åbo Akademi University 20500 Turku Finland reko.leino@abo.fi; Laboratory of Industrial Chemistry and Reaction Engineering, Åbo Akademi University 20500 Turku Finland

## Abstract

d-Fructose is an important starting material for producing furfurals and other industrially important chemicals. While the base-catalyzed and enzymatic conversion of d-glucose to d-fructose is well known, the employed methods typically provide limited conversion. d-Glucosone can be obtained from d-glucose by enzymatic oxidation at the C2 position and, subsequently, selectively hydrogenated at C1 to form d-fructose. This work describes an investigation on the hydrogenation of d-glucosone, using both chromatographically purified and crude material obtained directly from the enzymatic oxidation, subjected to filtration and lyophilization only. High selectivities towards d-fructose were observed for both starting materials over a Ru/C catalyst. Hydrogenation of the crude d-glucosone was, however, inhibited by the impurities resulting from the enzymatic oxidation process. Catalyst deactivation was observed in the case of both starting materials.

## Abstract 2

Investigation on the hydrogenation of both chromatographically purified and crude d-glucosone directly from its enzymatic production process to yield d-fructose.

## Graphical abstract

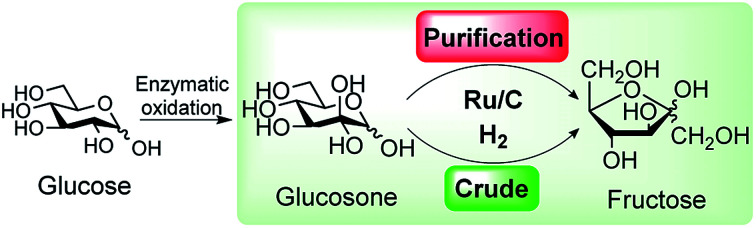

## Introduction

**Scheme 1:**
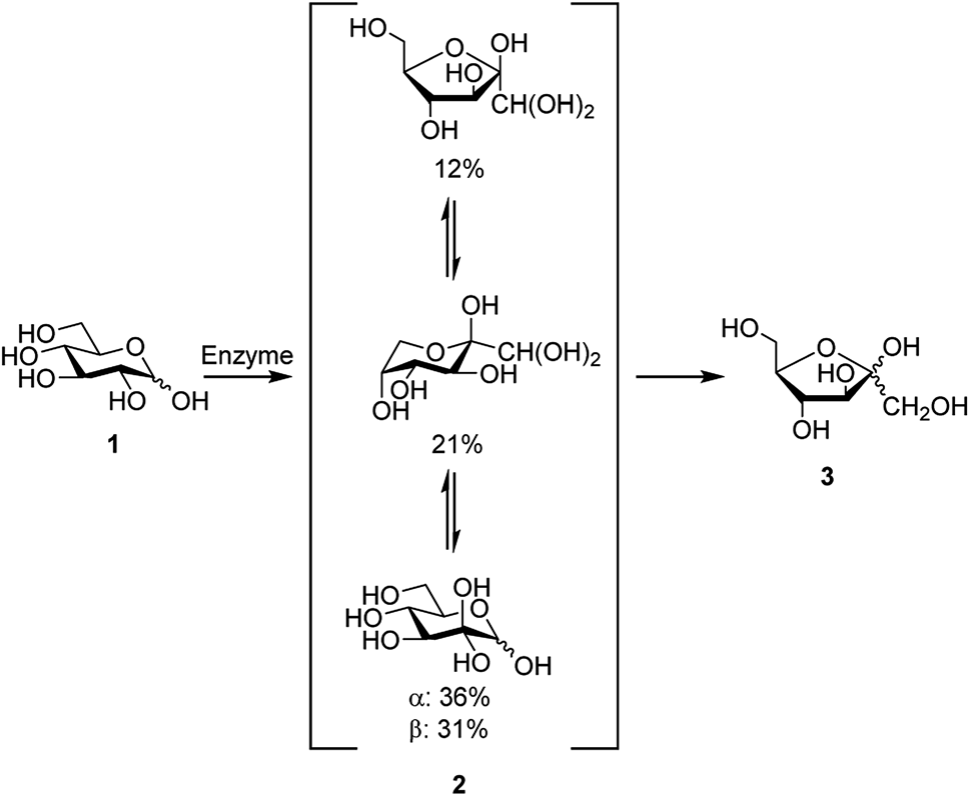
Formation of d-fructose (3) from d-glucose (1) *via*d-glucosone (2), showing the composition of 2 in aqueous solution.

Development of new synthetic methods for conversion of abundant carbohydrates, such as d-glucose, to other rare sugars and further to platform chemicals remains as a topical research field. d-Glucosone can be obtained from d-glucose by enzymatic oxidation at the C2 position (Scheme 1).^1–6^ In 6 h, 80% yield of d-glucosone can be achieved and in 10 h >95% is obtained.^6^ Similar to other carbohydrates, d-glucosone exhibits configurational equilibrium in aqueous solutions, where only hydrated forms of d-glucosone have been observed.^7^ Several hours are required to achieve equilibrium, the composition of which varies depending on temperature, pH, and concentration.^8^ While until now the use of d-glucosone has been limited due to its high price and limited availability, it has been shown recently, for example, that certain fine chemicals such as kojic acid could potentially be produced from this source.^6^ New enzymes, more suitable for industrial scale, are currently being developed and are likely to open the door for broader utilization of d-glucosone.^6^

Established methods exist already for producing various value-added chemicals, including furfurals, from d-fructose.^9,10^ The well-known base-catalyzed isomerization of d-glucose to d-fructose is performed in aqueous solutions using both homo- and heterogeneous catalysts.^11–16^ Such methods typically provide high selectivities, albeit at the expense of low yields due to unfavorable thermodynamics. The conventional process for synthesis of d-fructose involves glucose isomerase enzyme.^17^ The main challenges with this method are related to thermodynamic limitations, typically yielding a conversion of around 50%.^18^ Separation of the two sugars (*i.e.*, d-fructose and d-glucose) requires an expensive ion-exchange resin in the Ca^2+^ form, operating at 60–70 °C and resulting in up to 90% purity.^19^

An alternative path toward d-fructose is based on selective hydrogenation of d-glucosone.^20,21^ For example, Sun *et al.* have investigated the one-pot enzymatic oxidation of d-glucose to d-glucosone, followed by catalytic hydrogenation of d-glucosone to d-fructose.^21^ To ensure efficiency of the enzymatic reaction, mild conditions were required. Thus, long reaction times were necessary with the total time for converting d-glucose to d-fructose of 36 h. The mass balance and analysis of insoluble products were not addressed in their study. In addition substantial amounts of non-fructose carbohydrates were present meaning that purification is needed.

Efficient methods for conversion of d-glucose to d-fructose are essential for production of fine chemicals and biofuels from biomass with d-fructose being one of the key starting materials for further processing.^10,22–24^ A continuous method for d-glucosone production, allowing large quantities of d-glucosone to be accessed, combined with subsequent hydrogenation to produce pure d-fructose with minimal purification steps, would be a viable alternative to the existing methods.

Prior to a detailed technological and economical analysis of the two-step pathway involving enzymatic oxidation of d-glucose and the subsequent hydrogenation of d-glucosone, feasibility of the hydrogenation step using a real feedstock should be established. Purity of the feedstock and its influence on the catalytic performance has been seldom addressed in connection with biorefineries, as often in previous studies only model compounds have been used. However, it is well known that the presence of impurities can significantly deteriorate catalytic activity.^25^ In the present study, an evaluation of the effect of impurities leftover from the enzymatic process on the catalyst performance in d-glucosone hydrogenation is, therefore, addressed.

## Results and discussion

**Fig. 1:**
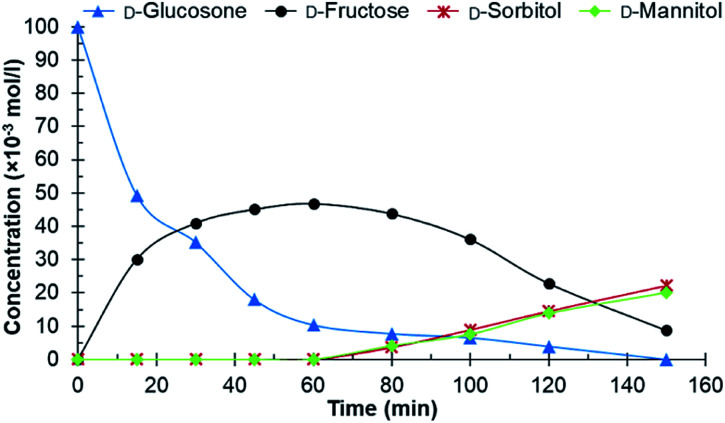
Conversion of purified d-glucosone in the batch reactor. Conditions: 100 ml 0.1 mol l^−1^ purified d-glucosone in H_2_O, 100 mg 4.6 wt% Ru/C catalyst, 20 bar H_2_, 110 °C.

Different catalysts were first screened in a batch reactor using purified d-glucosone as the starting material, before testing crude d-glucosone. The conditions used in the batch reactor were 20 bars H_2_ at 110 °C. These were selected based on prior investigations on carbohydrate hydrogenations.^26,27^ The crude d-glucosone, obtained from MetGen Oy, was only subjected to filtration through a 0.45 micron filter in order to remove enzyme particles after the enzymatic process where d-glucose is oxidized to d-glucosone.^6^ A portion of the crude d-glucosone was then further purified by column chromatography to obtain the purified d-glucosone used in the hydrogenation test reactions. The catalysts screened in the batch reactor were Ru/C, Ru/Al_2_O_3_, Ru on nitrogen doped carbon nanotubes (NCNT), Cu/SiO_2_, and RANEY® nickel. The Ru/Al_2_O_3_ catalyst showed traces of d-fructose after 180 min and a d-glucosone conversion of 51%, while the Cu/SiO_2_ catalyst exhibited traces of d-fructose, d-sorbitol, and d-mannitol, and a d-glucosone conversion of 66% after 180 min. The RANEY® nickel catalyst displayed 72% conversion after 120 min, resulting in 36% d-fructose yield without any d-sorbitol or d-mannitol detected. The Ru/NCNT catalyst gave 66% conversion after 180 min with 19% d-fructose yield and neither d-sorbitol nor d-mannitol. The most active catalyst was Ru/C, which then was selected for further experiments. After 1 h, 90% conversion of d-glucosone was observed (Fig. 1) with a subsequent conversion of d-fructose to d-mannitol and d-sorbitol.

**Fig. 2:**
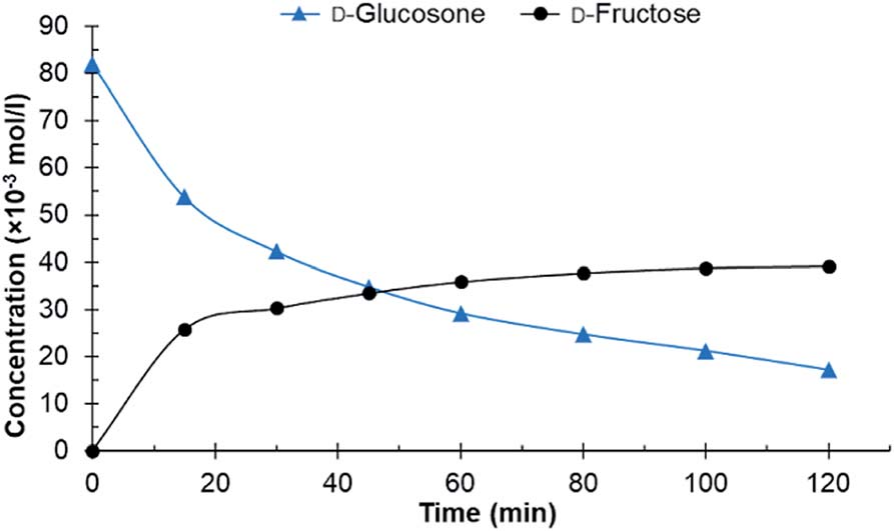
Conversion of crude d-glucosone in the batch reactor. Conditions: 100 ml 0.08 mol l^−1^ crude d-glucosone in H_2_O, 100 mg 4.6 wt% Ru/C catalyst, 20 bar H_2_, 110 °C.

After initial screening with purified d-glucosone, the crude d-glucosone was tested to investigate whether the impurities from the enzymatic oxidation of d-glucose to d-glucosone substantially influence the hydrogenation reaction. During the enzymatic process, NH_4_OH is added to maintain the pH at 6.6. The crude d-glucosone used contained *ca.* 18 wt% more impurities compared to the purified d-glucosone, as verified by HPLC analysis. Most likely, these impurities consist of salts and enzyme leftovers from the oxidation process. As seen from Fig. 2, conversion of crude d-glucosone remains incomplete after 120 min of the reaction and no further transformations of d-fructose to d-mannitol and d-sorbitol were observed. The results are consistent with catalyst deactivation or inhibition due to lower conversion of d-glucosone and a lower yield of d-fructose compared to the hydrogenation of purified d-glucosone.

The initial catalyst productivity, based on the conversion of d-glucosone to d-fructose, was 2.6 mol g^−1^ h^−1^ for the purified d-glucosone compared to 2.2 mol g^−1^ h^−1^ for crude d-glucosone. It has been established that salts and impurities significantly inhibit the catalyst activity during hydrogenation of carbohydrates.^25,27^ It was concluded in earlier work that NH_4_^+^ gave the most dominant deactivation among the salts tested during hydrogenation of carbohydrates on Ru/C catalyst.^27^ The added NH_4_OH in the enzymatic process of d-glucose oxidation forms NH_4_^+^, which most likely inhibits the catalyst in this work.

From both Fig. 1 and 2 it can be observed that the total concentration of analysed carbohydrates is decreasing. Using the purified d-glucosone, about 60% of the mass balance closure for carbohydrates was achieved after 60 min and for the crude d-glucosone this value is 91%, meaning that the impurities slow down the hydrogenation, but also the formation of side products. The formed side products could not be analysed using HPLC, indicating that they are insoluble in water and could be responsible for some type of deactivation, which was further investigated in the continuous reactor.

**Fig. 3:**
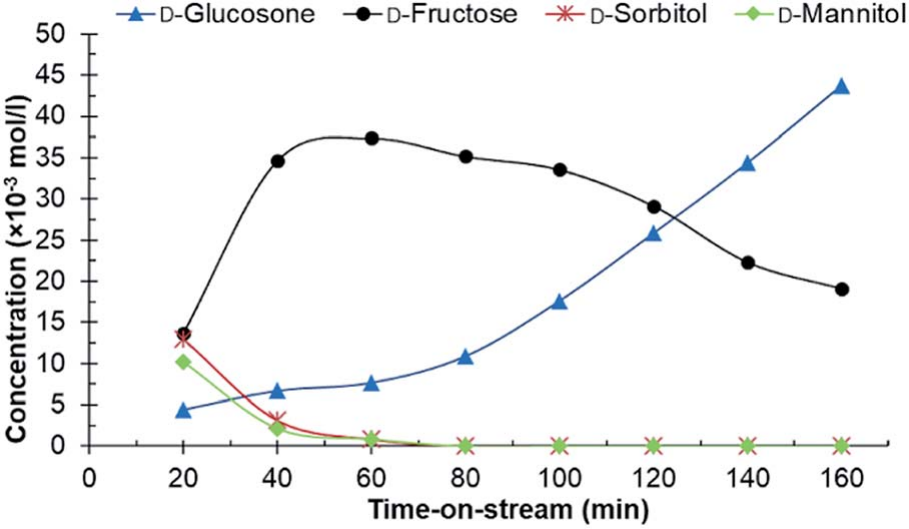
The conversion of d-glucosone in the continuous reactor. Conditions: 0.1 mol l^−1^ purified d-glucosone in H_2_O (0.5 ml min^−1^), 400 mg 0.7 wt% Ru/C catalyst, 20 bar H_2_ (25 ml min^−1^), 110 °C.

**Fig. 4:**
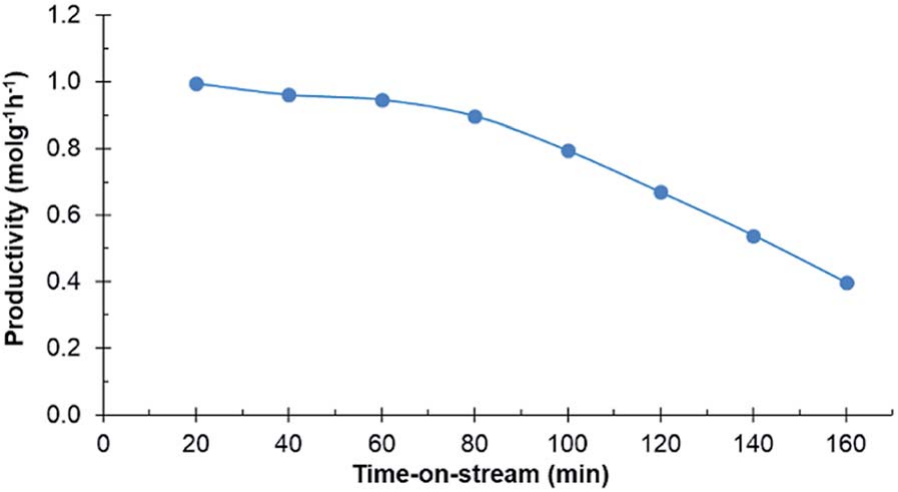
Productivity of the catalyst in the continuous reactor over time-on-stream. Conditions: 0.1 mol l^−1^ purified d-glucosone in H_2_O (0.5 ml min^−1^), 400 mg 0.7 wt% Ru/C catalyst, 20 bar H_2_ (25 ml min^−1^), 110 °C.

Experiments in the continuous reactor showed clear deactivation of the catalyst when the commercial 0.7% Ru/C was used as the catalyst (Fig. 3). The change in loading is justified considering potential industrial implementation of the catalyst. Extrudates of the commercial catalyst were first crushed to appropriate size prior to their use in the reactor operating in continuous mode. Conversion of d-glucosone steadily decreased with time on stream to 90% after 1 h, dropping to <50% after 140 min. After 60 min, neither d-mannitol nor d-sorbitol were produced, with d-fructose being the only product. As seen from Fig. 4, the catalyst productivity, based on d-glucosone conversion, starts to decrease significantly after 60 min and after 160 min the productivity is below 50% of the initially observed, consistent with catalyst deactivation. Leaching is not the underlying reason for the observed deactivation: only 0.05% of the total ruthenium used in the reactor had leached over 160 min, as determined by measuring the ruthenium concentration in the effluent solution by ICP. This confirms that the deactivation results from some other source. Similar to the batch reactor, a change in mass balance closure is observed during the continuous experiment from 47% to 62%, indicating that less side product is formed under catalyst deactivation conditions.

Next, deactivation was investigated in more detail by analysing the spent catalysts from the batch reactions. As shown in Table 1, the surface area of the Ru/C catalyst decreases significantly when using both purified and crude d-glucosone, to 12% and 14%, respectively, of the fresh catalyst surface area. Similarly, substantial changes in the pore volumes were observed when hydrogenating purified and crude d-glucosone, leading to a decrease of 42% and 58% respectively of the fresh catalyst. The smaller pores are blocked entirely due to fouling, increasing the apparent average pore size measured by nitrogen physisorption. Previously, it has been shown that d-glucosone has a tendency to oligomerize or polymerize at temperatures above 50 °C.^6^ The difference in mass balance closures probably results from the formation of polymers, which are deposited on the walls and adsorbed on the catalyst due to low solubility in water, resulting in fouling.

**Table 1:** Measured values of the spent and fresh catalysts from the batch experiments using N_2_ adsorption and desorption

Catalyst	Surface area (m^2^ g^−1^)	Pore volume (cm^3^ g^−1^)	Median pore width (Å)
Fresh catalyst	700	0.26	4.9
Spent catalyst with purified d-glucosone^a^	85	0.11	7.4
Spent catalyst with crude d-glucosone^b^	99	0.15	6.9

a Conditions: 100 ml 0.1 mol l^−1^ purified d-glucosone in H_2_O, 100 mg 4.6 wt% Ru/C catalyst, 20 bar H_2_, 110 °C, 150 min.

b Conditions: 100 ml 0.08 mol l^−1^ crude d-glucosone in H_2_O, 100 mg 4.6 wt% Ru/C catalyst, 20 bar H_2_, 110 °C, 120 min.

## Conclusions

As demonstrated in this study, selective hydrogenation of d-glucosone to d-fructose is possible using both purified and crude d-glucosone. Salts applied in the enzymatic oxidation of d-glucose to d-glucosone, such as NH_4_^+^, are the most probable catalyst inhibitors. Significant deactivation took place in the continuous reactor with the purified d-glucosone starting material. The main reason for catalyst deactivation is fouling, most likely due to oligomerization or polymerization of d-glucosone. Such deactivation could be clearly observed in the continuous reactor, where the productivity decreased significantly with the time on stream. When using the crude d-glucosone, 48% conversion to d-fructose was achieved after 2 h, while for the purified d-glucosone conversion of 42% was reached already after 1 h. Further investigations on minimization of the polymerization and fouling are needed to make this pathway from d-glucose to d-fructose viable for larger scale production.

## Experimental

d-Glucosone was obtained from MetGen Oy, produced by an earlier described method.^6^ The crude d-glucosone used in this work was obtained by filtration (0.45 micron) and freeze drying of the initially produced material obtained directly from the manufacturing process. Further purification was then carried out by column chromatography, using silica gel 60 (0.040–0.060 mm) as the stationary phase and MeOH/CH_2_Cl_2_ as the eluent. The 4.6% Ru/C catalyst had a Ru particle size of 2.5 nm and a catalyst size of <100 μm.^26^ The 3.6% Ru/NCNT catalyst with nitrogen doped carbon nanotubes as a support (Bayer Technical Services) had a Ru particle size of 3.3 nm and was crushed to a catalyst size of 125–250 μm.^26^ The 5.0% Ru/Al_2_O_3_ catalyst (Fluka) was used as such. The 20% Cu/SiO_2_ catalyst (BASF, catalyst H3-11) was crushed to <100 μm and used as such. The RANEY® nickel catalyst (Grace, RANEY® 3110) was likewise used as received. The 0.7% Ru/C catalyst (Engelhard) had a Ru particle size of 2.9 nm and a catalyst size of 250–355 μm.^26^ The catalysts are pre-reduced in hydrogen prior to storage. *In situ* pre-treatment of ruthenium catalysts prior to experiments at mild conditions was done to remove the oxide layer. The hydrogenation reactions were followed by high-performance liquid chromatography (HPLC) (HITACHI Chromaster HPLC) equipped with a refractive index (RI) detector. A Biorad HPX-87C carbohydrate column was used with 1.2 mM CaSO_4_ (0.2 ml min^−1^ flow rate) as the mobile phase. Temperature of the column and the detector were 80 °C and 40 °C, respectively. An accuracy within 3% was obtained with the HPLC. Leaching was investigated by measuring the concentration of ruthenium using a PerkinElmer ELAN 6100 DRC Plus, with a detection limit of 0.003 mg l^−1^. The fresh and spent catalysts were analyzed using Micrometrics MicroActive 3Flex 3500 instrument for surface area and pore size measurements, with an accuracy within 2%.

The mass balance is calculated as the ratio of the total carbohydrate concentration *C* to the initial one *C*_0_, *i.e. C*/*C*_0_.

The productivity was calculated for the batch reactor asΔ*C*/*t* × *m*_Ru_,where Δ*C* is the difference in the d-glucosone concentration, while for the continuous reactor the following expression was used for productivityΔ*ñ*/*m*_Ru_,where Δ*ñ* is the difference in d-glucosone molar flow at the inlet and outlet of the reactor.

### Batch reactor

**Fig. 5:**
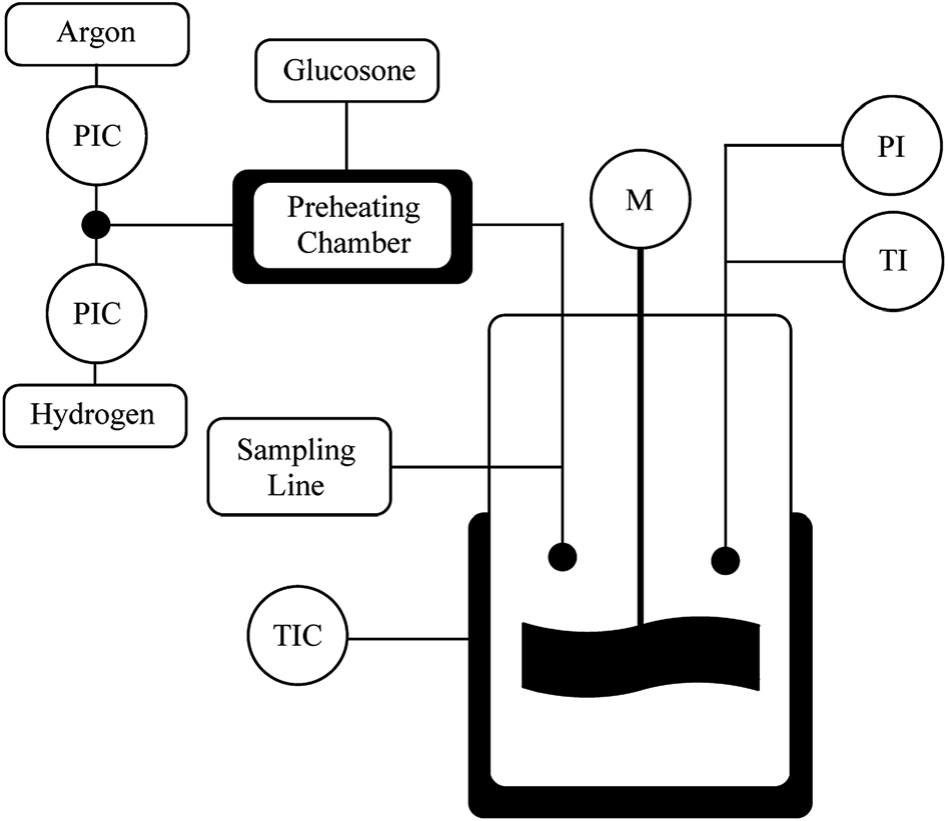
Batch reactor set-up for transformation of glucosone.

The catalyst (100 mg) was fed into the reactor (300 ml) and flushed with Ar and then with H_2_ (Fig. 5). The reactor was heated to 110 °C and the H_2_ pressure was kept at 4 bars for 1–1.5 h. d-Glucosone (2 g) was dissolved in 100 ml deionized water and heated to 80 °C. The d-glucosone solution was added to the reactor and the hydrogen pressure was raised to 20 bars. The reaction started with stirring at 1200 rpm. The samples were taken at desired intervals and analyzed by HPLC. Small catalyst particles were used under vigorous stirring (1200 rpm) to avoid internal and external mass transfer limitations.

### Continuous reactor

**Fig. 6:**
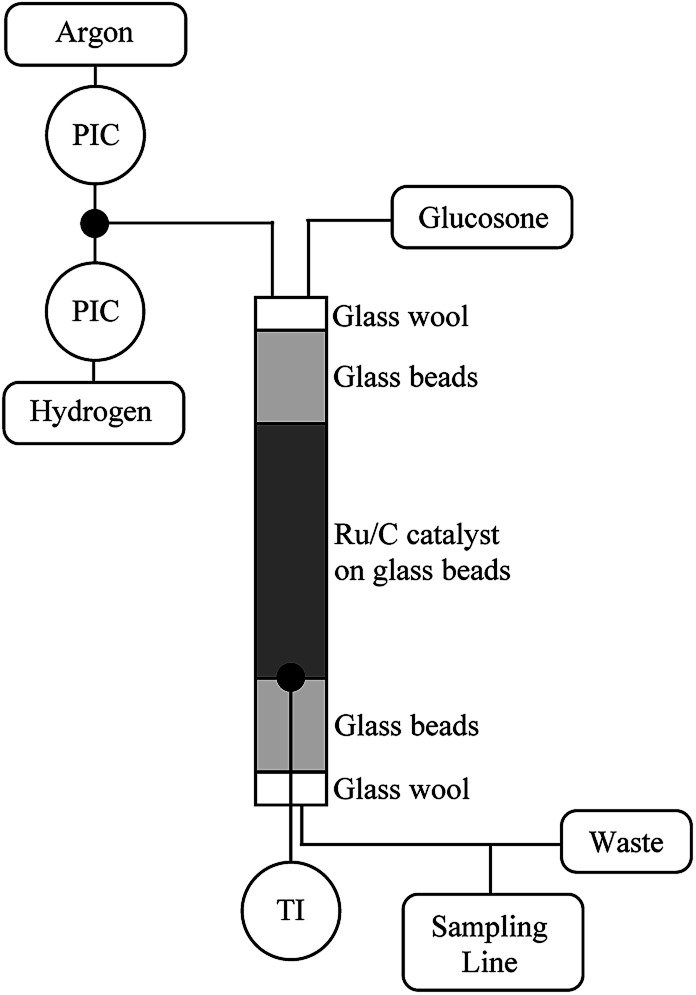
Continuous reactor set-up for transformation of glucosone.

The hydrogenation catalyst (400 mg) was mixed with glass beads (9.6 g) in a column through which the reaction solution was pumped at different rates. The reactor (diameter: 12.5 mm) was packed by first filling it with neat glass beads (425–600 μm) and then with the catalyst mixture and finally neat glass beads (Fig. 6). The substrate contact time to the catalyst was 2.4 min. The reactor was flushed with Ar and then with H_2_. The reactor was then heated to 110 °C and H_2_ pressure was raised to 20 bars. A 0.1 mol l^−1^d-glucosone solution was prepared and pumped into the reactor using an HPLC-pump (Agilent 1100 Quaternary pump) at a rate of 0.5 ml min^−1^. The H_2_ flow was adjusted to 25 ml min^−1^. The samples were taken every 20 min and analyzed by HPLC.

## Conflicts of interest

There are no conflicts to declare.

## Supplementary Material
